# Lung Ultrasound to Detect Pneumothorax in Children Evaluated for Acute Chest Pain in the Emergency Department: An Observational Pilot Study

**DOI:** 10.3389/fped.2022.812246

**Published:** 2022-03-10

**Authors:** Barbara Scialanga, Danilo Buonsenso, Simona Scateni, Piero Valentini, Paolo Maria Salvatore Schingo, Elena Boccuzzi, Maria Alessia Mesturino, Valentina Ferro, Antonio Chiaretti, Alberto Villani, Maria Chiara Supino, Anna Maria Musolino

**Affiliations:** ^1^Department of Emergency, Acceptance and General Pediatrics, Institute for Research and Health Care (IRCCS), Bambino Gesù Children's Hospital, Rome, Italy; ^2^Department of Woman and Child Health and Public Health, Institute for Research and Health Care (IRCCS), Fondazione Policlinico Universitario A. Gemelli, Rome, Italy; ^3^Dipartimento di Scienze di Laboratorio e Infettivologiche, Fondazione Policlinico Universitario A. Gemelli, Institute for Research and Health Care (IRCCS), Rome, Italy; ^4^Global Health Research Institute, Istituto di Igiene, Università Cattolica del Sacro Cuore, Rome, Italy; ^5^Department of Diagnostic Imaging, Institute for Research and Health Care (IRCCS), Bambino Gesù Children's Hospital, Rome, Italy

**Keywords:** pneumothorax, children, lung ultrasound, lung point, PNX, chest pain, pediatric, emergency department

## Abstract

**Background:**

Spontaneous pneumothorax is a relatively uncommon and poorly studied condition in children. While several protocols have been developed to evaluate the use of lung ultrasound for dyspneic adult patients in the emergency department, no specific guidelines are present for pediatric emergency physicians.

**Objectives:**

We prospectively analyzed children with acute chest pain and clinical suspicion of pneumothorax evaluated at the pediatric emergency department.

**Methods:**

We consecutively enrolled children aged 5–17 years presenting to the pediatric emergency department with clinically suspected pneumothorax based on sudden onset of acute chest pain. After clinical examination, all children underwent lung ultrasound followed by chest X-ray (reference standard). We enrolled 77 children, of which 13 (16.9%) received a final diagnosis of pneumothorax.

**Results:**

The lung point had a sensitivity of 92.3% (95% CI 77.8–100) and a specificity of 100% (95% CI 94.4–100) for the detection of pneumothorax. The “barcode sign” had a sensitivity of 100% (95% CI 75.3–100) and a specificity of 100% (95% CI 94.4–100) for the detection of pneumothorax.

**Conclusion:**

Lung ultrasound is highly accurate in detecting or excluding pneumothorax in children with acute chest pain evaluated in the pediatric emergency department. If pneumothorax is suspected, but the lung point is not visible, the barcode sign should always be sought as it could be a form of massive pneumothorax.

## Introduction

Spontaneous pneumothorax (PNX) is a relatively uncommon and poorly studied condition in children. A recent review reports an incidence of 3.41 per 100,000 patients younger than 18 years, while the incidence of secondary non-traumatic PNX in the pediatric population is not even established (1).

Although diagnosis of spontaneous PNX is often clinically suspected in the pediatric emergency department (pED) based on history and physical examination, spontaneous PNX is usually confirmed by chest X-ray (CXR), since computed tomography (CT) scan is not always available and bears a substantial dose of radiation exposure (2). Similarly, CXR has some limitations as it is not radiation free and the child needs to move from the pED room to the radiology unit, which might be difficult with unstable patients.

Point-of-care ultrasound (POCUS) and lung ultrasound (LUS) are now routinely used in most adult emergency departments (EDs): several protocols have been so far developed to evaluate point-of-care LUS to acutely dyspneic patients in the ED. In particular, the BLUE protocol developed by Liechtestein (2015) has proven effective in the diagnosis of pneumonia, edema, trauma complications, and PNX. In all adult studies on PNX, LUS showed high sensitivity and specificity (3–7). LUS is now considered non-inferior to CXR for the diagnosis of PNX when used by experienced physicians. While emergency guidelines for adults are available, there are no specific guidelines for pediatric emergency physicians despite the growing use of point-of-care lung ultrasound in pediatric EDs (8).

For these reasons, we performed this study to evaluate LUS accuracy in detecting PNX in children with acute chest pain in the pED.

## Methods

This prospective study was conducted between 1 July 2018 and 31 December 2019 in Bambino Gesù children hospital (Rome, Italy), a hospital with an annual census of about 65,000 ED visits. Approval from the ethics committee of our institution (protocol number 1564 OPBG2018) and informed consent were obtained. The study was not registered in a clinical trial registry.

After the initial clinical assessment, the same evaluating ED pediatrician performed LUS, always before performing CXR (reference standard).

The radiologist performing anteroposterior CXR and the pediatrician conducting lung ultrasound were blinded to the LUS and the CXR results, respectively.

LUS was carried out by three pediatricians who performed ultrasound scans in the pED for more than 5 years (9–13). They were aware of the patients' medical history and were the ones involved in diagnostic or therapeutic decisions. LUS was performed using a Sonosite MTurbo (Milan, Italy).

We consecutively enrolled patients aged 5 to 17 years presenting to the pED with clinically suspected PNX based on sudden onset of acute chest pain plus one of the following signs/symptoms: dyspnea, polypnea, diminished breath sounds, or hyperresonant percussion. Patients were enrolled only if one of three pediatricians were on duty.

Patients outside the age range; who refused to participate; with severe conditions requiring immediate life-saving procedures; with multiple contusions (such as car/motorbike crashing), pneumonectomy, chronic lung conditions (i.e., ciliary dyskinesia, cystic fibrosis, bronchopulmonary dysplasia, chronic respiratory failure, alpha 1-antitrypsin deficiency, pulmonary fibrosis, or congenital cystic adenomatoid malformation), cardiac abnormalities, and tracheal stenosis; and with known malignancies or subcutaneous emphysema were excluded from the study. Moreover, if the evaluating clinician suspected a specific disease (gastritis, gastroesophageal reflux, or musculoskeletal disorders), LUS and CXR were not deemed necessary and not performed.

All the enrolled patients initially underwent LUS, then the CXR, and subsequently, other diagnostic tests if necessary. In case one of these procedures was not performed, or if CXR was performed before LUS, the patient was excluded from the study.

### Measurements

LUS was performed using a portable ultrasound machine using a high-frequency linear probe (12 mHz). LUS was performed with patients in the supine position as described in the BLUE protocol using B-mode and M-mode settings; the LUS examination protocol was the same one we described previously (3, 9).

We carried out a semi-quantitative estimate of PNX size: in case of small PNX, the lung point was located over the anterior chest close to the parasternal line, while in large PNX, the lung point was located after the midaxillary line.

### POCUS Findings

As indicated by the literature, ultrasound signs of the PNX are the absence of lung sliding, absence of B lines, and the presence of a lung point and barcode in M-mode (10, 14–17). Physiopathologically, PNX is the detachment of visceral and parietal pleura by entrapped air in the pleural space. All ultrasonographic signs are due to the presence of air in pleural space.

Lung sliding represents a regular movement synchronized with respiration that occurs between the parietal and visceral pleura. In case of PNX, the air inside the pleural space prevents the display of the visceral pleura to the ultrasound and therefore “lung sliding” is not observed.

**Figure 1:**
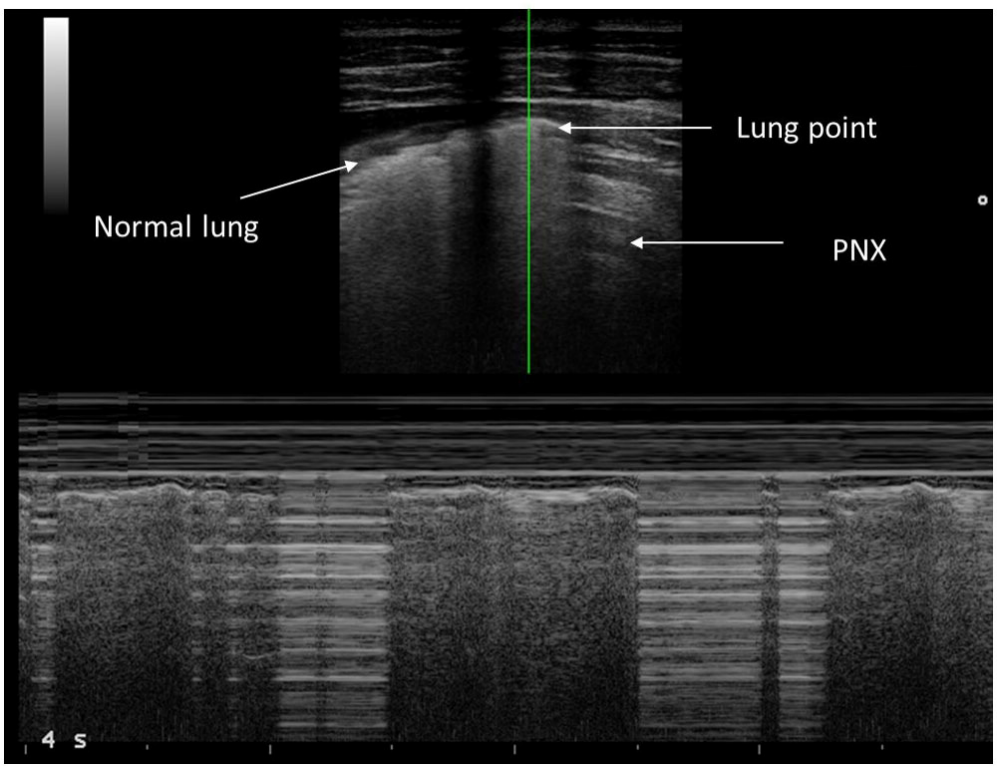
“Barcode sign” seen in M-mode.

Lung point represents the point between the absence of sliding and the resumption of normal sliding, which represents the physical limit of the PNX. Lung point is the point where the visceral pleura is again next to the parietal pleura without air interposition and slides with respiration (18). In M-mode ultrasound, the normal sliding generates seashore sing; in the case of PNX, you find the barcode sign, which is due to the absence of normal movement between the two pleuras (Figure 1).

The position of the lung point provided a semi-quantitative estimate of PNX size: in the case of a small PNX, the lung point was located over the anterior chest close to the parasternal line, while in a large PNX, it was located after the midaxillary line (17–19).

### Statistical Analysis

As this is a pilot study, no formal sample size planning has been considered, but at least 10 patients in total were planned to be enrolled in the study. Statistical analysis was performed using the SPSS software (IBM SPSS Statistics, version 24.0, Chicago, IL, USA). Values were expressed as means ± standard deviation (SD) for normally distributed continuous variables, median, and interquartile range (IQR) for data not normally distributed or number and percentage (%) for categorical variables. A *p* < 0.05 was considered statistically significant.

## Results

### Study Population

Seven hundred eighty-four children consecutively presented to our pED with a primary complaint of acute chest pain during the study period. Ninety-nine were excluded because they did not meet the inclusion criteria. One hundred thirty received a specific clinical diagnosis that, according to the evaluating physician, precluded the use of LUS and CXR. Of the remaining 555, 478 did not undergo LUS because operators were not available; of these, only two received a final diagnosis of PNX. In total, 77 children received LUS. Thirty of 77 (39%) children had interstitial lung disease (e.g., viral bronchitis); 20/77 (26%) had pneumonia with or without pleural effusions; 7/77 (9.1%) had thoracic trauma; 7/77 (9.1%) had a final diagnosis of myocarditis/pericarditis; and 13/77 (16.9%) received a final diagnosis of PNX. Among the 77 children, 44 (57.1%) were male with a median age of 10 years and 3 months (IQR 6 years and 9 months−14 years and 2 months). During the ED visit, 36 (46.8%) patients presented with dyspnea with a mean saturation of 98 ± 2% in ambient air.

**Figure 2:**
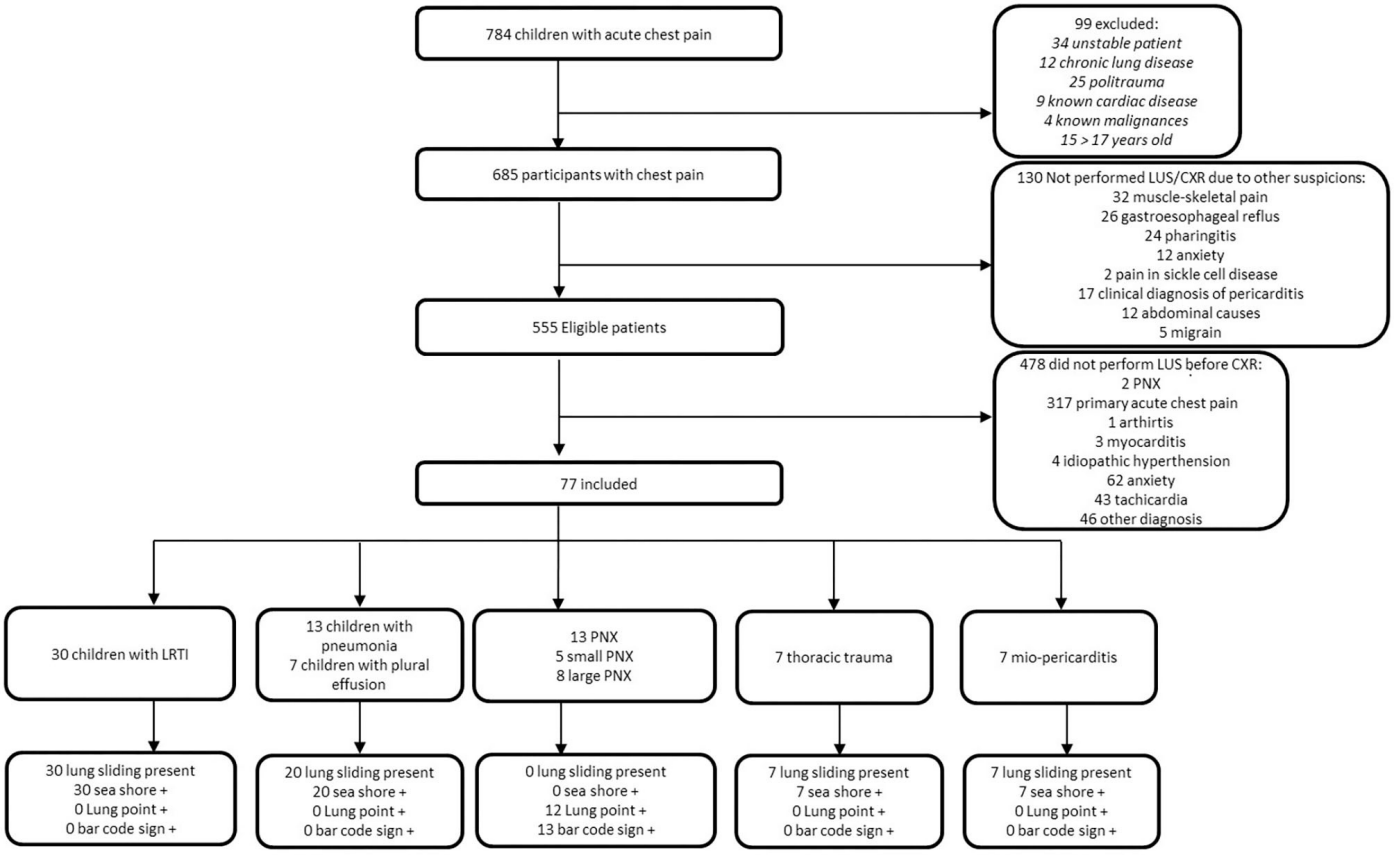
STARD diagram of flow of participants through the study.

Figure 2 shows the STARD diagram of the flow of participants through the study.

### Population With PNX

The demographic and clinical characteristics of the study population according to final diagnosis are reported in Table 1. Among the 13 patients with PNX, eight (61.5%) were males with a median age of 16 years and 1 month (IQR 15–16 years and 7 months), and only two (15.4%) presented in ED with dyspnea. The mean oxygen saturation was 99 ± 1% in ambient air, the heart rate 97 ± 15 beats per minute, and the respiratory rate 20 ± 2 breaths per minute.

**Table 1:** Demographic and clinical characteristics of the study population dived in according to final diagnosis.

	**LTRI (n**°**30)**	**Pneumonia with/without pleural effusion (n**°**20)**	**PNX (n**°**13)**	**Thoracic trauma (n**°**7)**	**Mio-pericarditis (n**°**7)**
Age, years-months (IQR)	8 years and 8 month (IQR 6 years and 3 months−10 years and 1 month)	8 years and 6 month (IQR 6 years and 2 months−11 years and 3 month)	16 years and 1 month (IQR 15 years−16 years and 7 months)	15 years and 4 month (IQR 12years and 3 months−16 years and 5 months	9 years and 4 month (IQR 8 years and 5 months−10 years and 3 months
Sex, males n° (%)	13 (43.3)	8 (40)	8 (61.5)	6 (85.7)	5 (71.4)
Heart Rate, bpm (SD)	109 (± 17)	113 (± 14)	97 (± 15)	104 (± 13)	110 (± 9)
Respiratory Rate, breath/min (SD)	24 (± 3)	23 (± 3)	20 (± 2)	22 (± 2)	23 (± 2)
Mean SaO2 in ED, % (SD)	97 (± 1)	97 (± 2)	99 (± 1)	98 (± 1)	99 (± 0)
Dyspnea in ED, n° (%)	12 (40)	9 (45)	20 (15.4)	7 (100)	6 (85.7)

In five (38.5%) patients, we diagnosed a secondary PNX; in two, uncontrolled allergic asthma; in two, suspected collagenopathy; and in one, smoking (cannabis). In four children (30.8%), a secondary episode of PNX was diagnosed; in three cases (23.1%), patients reported a history of chest pain without a specific diagnosis. Eleven patients (84.6%) were admitted for clinical observation; and four of them (30.8%) needed a chest tube placed.

All cases of PNX were confirmed by CXR; there were no cases of uncertainty about the presence/absence of PNX on CXR and LUS. Nine of them (69.2%) subsequently also underwent a chest CT, and lung abnormalities (bullae) were found in four (30.8%) cases that were not described by LUS.

### LUS Findings

In all 13 patients, LUS showed the “barcode sign,” while in 12 (92.3%) patients, there was a lung point, giving a diagnosis of PNX.

In five (38.5%) cases, the PNX was small, and the lung point was identified on the anterior chest surface between the parasternal and anterior axillary line; in two cases, the lung point was in the right hemithorax, while in three cases, it was in the left hemithorax. In eight (61.5%) cases, the PNX was considered large; the lung point was identified on the lateral surface of the thorax after the midaxillary line in seven cases, in the right hemithorax in three cases, and in the left hemithorax in four cases.

The lung point had a sensitivity of 92.3% (95% CI 77.8–100*)* and a specificity of 100% (95% CI 94.4–100), a positive predictive value of 100% (95% CI 73.5–100), and a negative predictive value of 98.4% (95% CI 91.6–100*)* for the detection of PNX. We did not find the lung point in one child with massive PNX with complete lung collapse. The “barcode sign” had a sensitivity of 100% (95% CI 75.3–100) and a specificity of 100% (95% CI 94.4–100), a positive predictive value of 100% (95% CI 75.3–100), and a negative predictive value of 100% (95% CI 94.4–100) for the detection of PNX.

## Discussion

In our study, we enrolled 77 children evaluated in the ED for acute chest pain. Our study shows the high accuracy of LUS in detecting PNX in pediatric patients evaluated in the pED for acute chest pain. While the role of LUS for PNX detection is widely described in adult patients, to our knowledge, this is the first prospective description in children evaluated in a pED for acute chest pain.

So far, studies on adults showed that LUS had a sensitivity of 78.6% (95% CI 68.1–98.1) and a specificity of 98.4% (95% CI 97.3–99.5), while CXR had a pooled sensitivity of 39.8% (95% CI 29.4–50.3) and a specificity of 99.3% (95% CI 98.4–100) (17, 20).

In our cohort, the lung point had a sensitivity of 92.3% (95% CI 77.8–100) and a specificity of 100% (95% CI 94.4–100) for the detection of PNX.

The “barcode sign” had a sensitivity of 100% (95% CI 75.3–100) and a specificity of 100% (95% CI 94.4–100) for the detection of PNX. Lung point was negative in one child with massive PNX with complete lung collapse, suggesting that when PNX is suspected, both signs must be looked for to confirm or exclude PNX.

The 100% sensitivity and specificity of the results of our pediatric patients can be explained by two factors. First, pediatric chests are easily evaluable with high-resolution linear probes (we used 12 mHz) due to their smaller sizes (having less muscles and fat); thus, the detection of the lung point is more easily obtained for pediatric chests than for adult chests. Second, our team is composed of pediatricians highly skilled and experienced in LUS (9–13). In fact, although current literature suggests that a 2-h training is sufficient to accurately train emergency physicians in detecting PNX, we think that such data are quite optimistic and can lead to wrong LUS examination (21). In daily clinical practice, the evaluation of non-compliant patients (a frequent situation in pediatric practice) may lead to false-positive or false-negative results; therefore, a high grade of experience along with clinical suspicion are both critical factors when performing LUS. For example, some authors questioned the specificity of the lung point, but they have been debunked by experts in LUS that showed how their false-positive/false-negative results were due to mistakes in performing LUS (22–26). In addition, a recent study on traumatic PNX in children showed a 45.5% sensitivity; however, LUS was performed by ultrasound technicians with a convex 2–5 MHz probe: these two factors can, in our opinion, explain the unexpected poor results of Vasquez et al. (27).

Evaluation of PNX requires knowledge of several LUS artifacts including lung sliding, lung point, B-lines, and M-mode (Figure 1) (28). The presence of lung sliding rules out PNX in the specific area of LUS examination (29). However, the absence of lung sliding does definitely indicate PNX. For example, pleurodesis, chronic lung disease, or severe parenchymal disease or bronchial occlusion can eliminate lung sliding, although these situations are rare in children. Therefore, when lung sliding is not looked for, a lung point must be found to confirm PNX (17). The lung point is 100% specific for PNX in adult studies (14). In our study, all children with PNX had a barcode sign and absence of lung sliding in the PNX area, while lung point was present in all but one case of massive PNX, confirming, for the first time to our knowledge, that the adult findings of PNX can be translated to children. The absence of lung point in a case of massive PNX is an important finding that highlights the need of always looking for all ultrasound signs when a PNX needs to be ruled out/in.

The high accuracy of LUS for PNX in our study can have practical implications. Pediatric studies have shown the high rate of CXRs performed by emergency pediatricians evaluating children with acute chest pain (30, 31). Considering the ability of LUS in detecting PNX, the high negative predictive value for PNX, and the available data on the accuracy of LUS in detecting PNX, the routine application of LUS can allow a high reduction of CXR-related costs and radiation exposure for the evaluation of acute chest pain in the pED.

We have performed a chest CT scan after CXR to detect predisposing lung conditions (e.g., bullae) because they are not recognizable by either CXR or LUS, and this is one of the major limits of LUS in comparison with CT scan. The lung pattern of the bullae is not yet well-established, although a recent study on congenital lung malformation described cystic lung lesions in neonates (32).

All patients were invited at the time of discharge to return to our ED after 48 h in case of persistence of symptoms or sooner in case of clinical deterioration. None of the patients enlisted and discharged returned to our ED.

The low number of children with acute chest pain evaluated by LUS is a limitation of our study; this was due to the fact that in our pED, we have only three highly trained LUS sonographers who performed the study and this has caused the exclusion of 86% of the eligible patients. This could have distorted the consecutive inclusion of the patients. In our study, only 0.4% of patients excluded, because no operators were on duty, had a PNX vs. 16.9% among the included patients. This difference is based on coincidence.

Another limitation of our study is that four patients did not undergo a chest CT. This is due to the clinical conditions of the patients, where after the recognition of the extended PNX with ultrasound, the patients were taken directly to the operating room for the positioning of the chest tube.

## Conclusion

In conclusion, our study showed the ability of LUS in detecting and excluding PNX in children evaluated in the pED for acute chest pain. Further studies on a larger number of children are needed to confirm our findings in order to allow routine application of LUS for pediatric acute chest pain in the ED.

## Data Availability Statement

The raw data supporting the conclusions of this article will be made available by the authors, without undue reservation.

## Ethics Statement

The studies involving human participants were reviewed and approved by 1564OPBG2018. Written informed consent to participate in this study was provided by the participants' legal guardian/next of kin.

## Author Contributions

MCS, DB, and AMM: conception and research design. SS, PV, and AC: data collection. PMSS, BS, MAM, EB, and VF: data analysis, interpretation, and drafting the article. All authors approved the final manuscript as submitted and agree to be accountable for all aspects of the work.

## Conflict of Interest

The authors declare that the research was conducted in the absence of any commercial or financial relationships that could be construed as a potential conflict of interest. The handling editor declared a past collaboration with one of the authors AC.

## Publisher's Note

All claims expressed in this article are solely those of the authors and do not necessarily represent those of their affiliated organizations, or those of the publisher, the editors and the reviewers. Any product that may be evaluated in this article, or claim that may be made by its manufacturer, is not guaranteed or endorsed by the publisher.
